# Refining Atmosphere Profiles for Aerial Target Detection Models

**DOI:** 10.3390/s21217067

**Published:** 2021-10-25

**Authors:** Robert Grimming, Patrick Leslie, Derek Burrell, Gerald Holst, Brian Davis, Ronald Driggers

**Affiliations:** 1 College of Optics and Photonics, University of Central Florida, 4304 Scorpius Street, Orlando, FL 32816, USA; 2 Wyant College of Optical Sciences, University of Arizona, 1630 East University Boulevard, Tucson, AZ 85721, USA; leslieps@email.arizona.edu (P.L.); derekburrell@email.arizona.edu (D.B.); rdriggers@arizona.edu (R.D.); 3 JCD Publishing Co., Oviedo, FL 32765, USA; jerry@jcdpublishing.com; 4 CAE USA (Link), 2200 Arlington Downs Road, Arlington, TX 76011, USA; Brian.Davis@caemilusa.com

**Keywords:** infrared detection, atmospheric radiation, path radiance, sky temperatures

## Abstract

Atmospheric path radiance in the infrared is an extremely important quantity in calculating system performance in certain infrared detection systems. For infrared search and track (IRST) system performance calculations, the path radiance competes with the target for precious detector well electrons. In addition, the radiance differential between the target and the path radiance defines the signal level that must be detected. Long-range, high-performance, offensive IRST system design depends on accurate path radiance predictions. In addition, in new applications such as drone detection where a dim unresolved target is embedded into a path radiance background, sensor design and performance are highly dependent on atmospheric path radiance. Being able to predict the performance of these systems under particular weather conditions and locations has long been an important topic. MODTRAN has been a critical tool in the analysis of systems and prediction of electro-optical system performance. The authors have used MODTRAN over many years for an average system performance using the typical “pull-down” conditions in the software. This article considers the level of refinement required for a custom MODTRAN atmosphere profile to satisfactorily model an infrared camera’s performance for a specific geographic location, date, and time. The average difference between a measured sky brightness temperature and a MODTRAN predicted value is less than 0.5 °C with sufficient atmosphere profile updates. The agreement between experimental results and MODTRAN predictions indicates the effectiveness of including updated atmospheric composition, radiosonde, and air quality data from readily available Internet sources to generate custom atmosphere profiles.

## 1. Introduction

In the infrared systems application space, detecting dim unresolved targets against the sky path radiance is critical. This task encompasses both military and commercial systems such as long-range aircraft detection in a passive mode (offensive infrared search and track (IRST), as well as an increasingly important function of short- and long-range drone detection. In the past few years, drones have disrupted airport operations and used as a bomb to attack presidents [1,2]. The design of systems for detecting these drones requires a deep understanding of atmospheric path radiance (usually the background of the drone image). Detection with infrared cameras provides potential advantages because of a drone’s low radar signatures and use during low-light scenarios. Due to their size, these targets may be a few pixels or even unresolved at a kilometer away. For any imaging system, a drone is detected when there is sufficient contrast with the background, in this case, the sky.

When developing contrast enhancement procedures, convolutional neural networks (CNNs), object detectors, etc., the goal is to improve the probability of detection [3,4,5,6,7,8,9]. Therefore, characterizing and modeling the sky background is essential.

Today, a relatively inexpensive uncooled microbolometer-based camera and expensive cooled infrared systems based on InSb or HgCdTe detectors can be used for detecting drones. In either case, the output is a two-dimensional array of grayscale values. In addition, these cameras may be radiometrically calibrated to provide a radiance value (W/cm^2^/sr) or equivalent blackbody temperature (K) of the source. The latter is sometimes preferred because it is easier to relate to, and midwave infrared (MWIR) and longwave infrared (LWIR) camera sensitivities are expressed in noise equivalent temperature difference (NETD) that range in the tens of mK.

**Figure 1:**
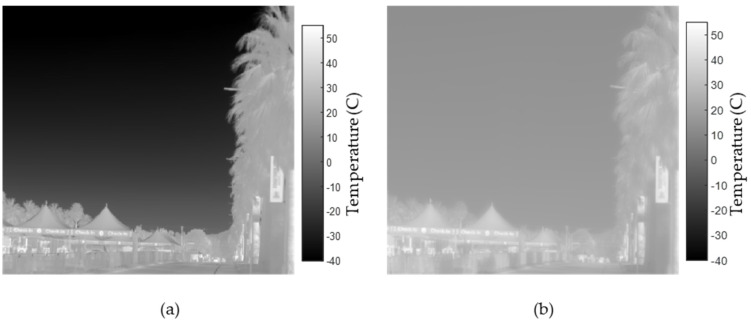
Sky temperature map of Tucson, Arizona, measured in degrees Celsius for the horizon to approximately 20° elevation near midday on 25 February 2021: (**a**) LWIR image taken with FLIR T1020; (**b**) MWIR image taken with TELOPS M1k.

The sky appears cold on a clear day with low humidity, quickly reaching −40 °C at an elevation angle of 15° in LWIR, as shown in Figure 1. Conversely, the same sky appears much warmer in the MWIR at more than 10 °C. Path radiance is generated due to the thermal emission of the atmosphere along a line of sight. Depending on the depth of the atmosphere and its contribution at different altitudes, the path radiance varies with elevation angle for a ground-based sensor.

These differences, as seen in Figure 1, exemplify why a constant sky background would poorly model a system’s ability to detect a small aerial target. In terms of radiance, the contrast between a drone and the sky depends on the atmospheric conditions according to Equation (1). The spectral target contrast, Δ*L*(*λ*), in radiance is

(1) $$\begin{array}{cc} \Delta L\left(\lambda \right)=\left[\tau_{ATM}\left(\lambda \right)L_{TGT}\left(\lambda \right)+L_{FRGD}\left(\lambda \right)\right]-L_{SKY}\left(\lambda \right) & \frac{W}{cm^{2}\cdot sr\cdot \mu m} \end{array}$$

The target radiance, *L_TGT_*(*λ*)*,* is first reduced by the atmospheric transmission, *τ_ATM_*(*λ*). It is then affected by the foreground path radiance, *L_FRGD_*(*λ*)*,* in front of the target. This quantity finally contrasts with the background or sky path radiance, *L_SKY_*(*λ*), between the camera and space. The atmospheric transmission and sky path radiance must be known to predict the probability of detection with programs such as NVThermIP or NVIPM [10]. Since it is not practical to measure every atmospheric condition to obtain these values, radiation transfer software that models different atmospheric conditions is used instead.

MODTRAN remains the industry standard for evaluating the radiative transport equations to obtain spectral transmission and path radiance values along a specified line of sight for a specific atmosphere profile [11]. Current versions of MODTRAN provide six standard atmosphere profiles based on geographic latitude and season. It also includes tools to create custom profiles by incorporating location-based historic radiosonde averages or daily collected radiosonde data along with applying different aerosol configurations to the custom layers.

### 1.1. Related Work

Determination of path radiance and atmospheric transmission with MODTRAN’s standard profiles has often led to differences compared with measured data. One method to reduce the difference between predicted and measured values is to carefully match local radiosonde to the six standard profiles and use the best match and correct the sun’s positioning about the sensor [12,13]. Various other publications cite using MODTRAN with the Navy Maritime Aerosol Model to improve results [14,15]. In these cases, these methods provide a closer result when measurements are taken if the atmospheric conditions are close to annual averages but not extremes.

Several other radiation transfer model software programs exist, such as 6SV, LibRadtran, and OPEC [16]. In the comparison study performed in [16], different aerosol types, gas concentrations, and vertical profiles were compared. In general, the programs provide results differing by 10–20% using similar US Standard 1976 atmosphere profiles. None of the results were compared with measured broadband results.

In [10], the authors provide data to “illustrate the importance of modeling atmospheric effects”. In this work, a comparison is made between MODTRAN’s standard atmosphere profiles and atmosphere profiles based on localized weather data for various US cities. They concluded that the standard atmospheres are acceptable for the extremes, i.e., MidLat summer vs. MidLat winter, but are insufficient for performing reliable probability of detection calculations at a specific location, date, and time. In addition, the authors, similar to many others, note that to take advantage of MODTRAN’s full capabilities, one must be an experienced user.

### 1.2. Contributions

The focus of this paper is to consider the customization of an atmosphere profile in MODTRAN to obtain reliable predictions for path radiance and equivalent blackbody temperatures of the sky for a specific location, date, and time. Using readily available Internet sources in our method, we demonstrate the ease and advantages of using custom atmosphere profiles in modeling. Furthermore, since MODTRAN Version 6 incorporated an application programming interface (API), we also provide a simple MATLAB interface script demonstrating how to customize atmosphere profiles and incorporate available atmospheric data. The contributions of this work are as follows:

1. A detailed description of the results obtainable by adding location, date, and time-specific data to a custom atmosphere profile in MODTRAN;
2. Validation of MODTRAN’s predicted sky path radiance values, compared with measured data obtained with an uncooled LWIR and a cooled MWIR broadband camera;
3. Recommendations for customizing MODTRAN atmosphere profiles for specific tasks, including predictive modeling and data analysis for detecting and tracking drones against a sky background.

For convenience and readability, measured and predicted values were converted to equivalent blackbody temperatures throughout this article. Conversion to other radiometric quantities is possible, but it should be noted that the equivalent blackbody temperatures reported in this article are specific to the cameras used and their manufacturer-provided relative spectral response.

## 2. Materials and Methods

Each example presented in this article represents a particular dataset of measured sky path radiance values converted to equivalent blackbody temperatures. Example 1 was performed in Cape Canaveral, Florida (28.65 N Lat., 80.67 W Long.) on 26 October 2020 at 08:00 local time (12:00 UTC). This location is inside the Cape Canaveral National Park and is flat, rural terrain consisting of marshes with small trees and grass. Example 2 was performed in Tucson, Arizona (32.23 N Lat., 111.01 W Long.) on 31 January 2021 at 18:30 local time (00:30 UTC). Finally, Example 3 was performed at the same location in Tucson, Arizona, on 5 March 2021 at 19:00 (00:00 UTC). The Tucson location was in the Saguaro National Park and overlooked hilly terrain that is rocky with small shrubs. Sky measurements were predominantly taken during periods of clear sky, and we attempted to match data to reported radiosonde balloon launches. Measurements were taken in a 90° arc, avoiding direct viewing of the sun and clouds.

### 2.1. Custom Atmosphere Examples

Internet databases readily provide daily, monthly, or annual readings of atmospheric composition [17,18]. The main constituents that affect infrared radiation transfer in the atmosphere include water vapor, ozone (O_3_), carbon dioxide (CO_2_), nitrous oxide (N_2_O), methane (CH_4_), and chlorofluorocarbons [19,20]. To a lesser extent, sulfur dioxide (SO_2_), nitrogen dioxide (NO_2_), and carbon monoxide (CO) also have absorption peaks in the infrared. The corresponding absorption peaks in the infrared for these components are shown in Table 1. Ground-level concentrations of O_3_, CO, NO_2_, and SO_2_ are reported as air quality indices [21,22].

Most of MODTRAN’s default atmospheric data are outdated, as they are based on research and measurements from the 1970s [11]. MODTRAN’s Atmosphere Generator Toolkit (AGT) indeed updates some of the scaling factors for cross-sectional species but uses values from 2002. The MODTRAN 6 User’s Guide provides 2015 values. The AGT can access historical daily and monthly radiosonde averages provided by NOAA’s NCEP/NCAR Reanalysis database for 2.5° × 2.5° gridded sectors on Earth. These data include vertical profiles to approximately 30 km for pressure, temperature, and water vapor. The AGT also allows for importing twice-daily radiosonde data from the University of Wyoming’s weather database, which provides the same vertical profiles as the NCEP/NCAR Reanalysis database. For the upper atmosphere, from 30 km to 100 km, the AGT selects the closest match of the six default profiles based on the lower atmosphere data.

The US EPA’s Air Quality monitoring stations and others located worldwide report ground-level concentrations [21]. In general, the stations provide concentrations of O_3_, CO, NO_2_, and SO_2_ along with small and large particulate matter concentrations. Ground-level concentrations usually apply to the first 3 km of the atmosphere above sea level before becoming uniformly mixed due to higher altitude winds [19].

A custom atmosphere profile can be generated using the AGT, followed by updating several other atmosphere values. Table 2 shows the values for the three examples used in this article.

For all three examples, the Rural aerosol model was used. As the most basic aerosol model for the ground layers used by MODTRAN in the Continental US, Rural consists of 70% small particles such as water-soluble substances and organics and 30% large particles such as dust [11]. The Urban aerosol model adds soot that accounts for 20% soot (carbon-based substances), and the remaining 80% is the Rural concentrations. For each of our examples, the visibility each day was reported as “greater than 10 miles (16 km)” by weather sources, so the default Rural aerosol model was used with a 16 km range.

### 2.2. Cameras

**Figure 2:**
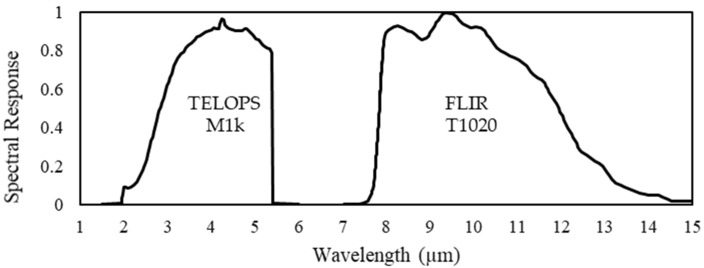
Relative spectral response of TELOPS M1k and FLIR T1020 cameras. The spectral response is representative and can vary with the optical package or camera configuration.

The cameras used to measure sky temperatures were a FLIR T1020 Thermal Camera for LWIR and a TELOPS Fast-IR M1k for MWIR. For each camera, its relative spectral response provided by the manufacturer is shown in Figure 2.

The camera’s relative spectral response, *R_CAMERA_*(*λ*), is used with the Planck’s radiation law to determine an equivalent blackbody temperature according to

(2) $$\begin{array}{cc} \int_{\lambda_{1}}^{\lambda_{2}}R_{CAMERA}\left(\lambda \right)L_{BB}\left(T,\lambda \right)d\lambda =\int_{\lambda_{1}}^{\lambda_{2}}R_{CAMERA}\left(\lambda \right)L_{SKY}\left(\lambda \right)d\lambda & \frac{W}{cm^{2}\cdot sr\cdot \mu m} \end{array}$$

In Equation (2), there is a specific equivalent blackbody temperature, *T*, for the spectral blackbody radiance, *L_BB_(T,λ*), to satisfy the equality when a sky path radiance, *L_BB_(T,λ*), is generated by MODTRAN. Radiometrically calibrated cameras provide a lookup table that relates the equivalent blackbody temperature to the pixel’s grayscale value. When the camera is not radiometrically calibrated, a simplified process to create a signal transfer function (SITF) for relating the pixel’s grayscale value, *U_D_*, to an equivalent blackbody temperature can be used. The process consists of viewing a series of blackbody targets at different temperatures and recording the pixel values. The results are then approximated using Equation (3) [24].

(3) $$\begin{array}{cc} T=\frac{B}{\ln\left(\frac{R}{U_{D}-O}+F\right)} & K \end{array}$$

The value *B* represents the exponential coefficient in Planck’s radiation law that includes Planck’s constant, Boltzmann’s constant, the speed of light, and effective wavelength, or *B = hc/kλ_EFF_*. The value *R* represents the average spectral response of the camera, while *F* adjusts the curve for the nonlinear response of the detectors. *O* is an offset to account for light outside the camera’s field of view that falls on the detectors, such as from the camera body or cold shield.

In addition, any factors that may affect the spectral response of the cameras, such as the sensor’s operating temperature and responsitivity variation from camera to camera, may not be captured when Equation (2) is used to calculate the equivalent blackbody temperature.

### 2.3. Building a Custom Atmosphere

**Figure 3:**
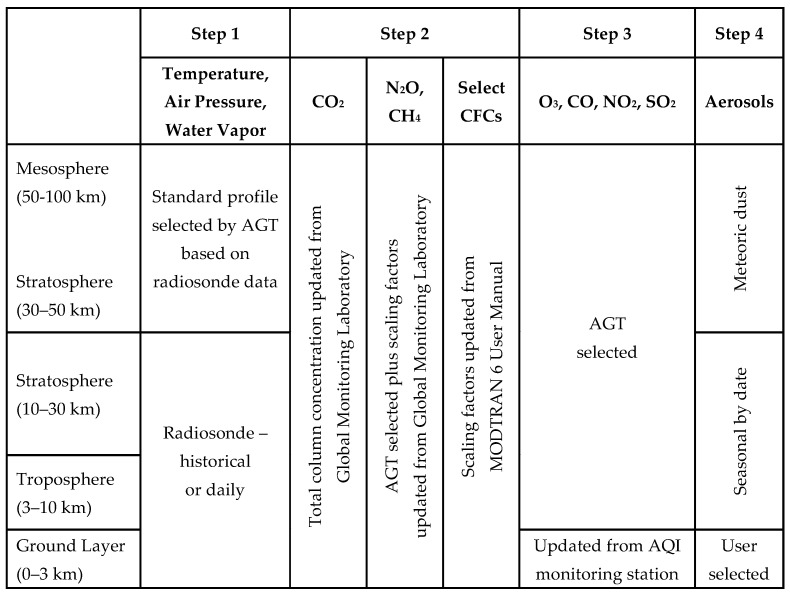
Custom atmosphere workflow overview.

The C/C++ code that is part of MODTRAN’s API can be called by MATLAB using the *MEX* command. A *MEX* command treats the C/C++ code as a subroutine or function for Matlab, allowing scripting and parameter manipulation. The JavaScript Object Notation (JSON) text file structure is compatible with MATLAB and MODTRAN, making it easy to transfer structured variables between the applications without parsing traditional MODTRAN input and output tape files. Figure 3 shows the workflow for creating a custom atmosphere and is reflected in the Supplementary Materials.

First, the AGT was used to modify the lower atmosphere’s temperature, air pressure, and water vapor profiles. For creating custom atmospheres with the AGT, command-line statements were executed according to MODTRAN 6 User Manual. The AGT accessed the historical averages of radiosonde data from the NCEP/NCAR Reanalysis database or processes a University of Wyoming weather database file. Since the radiosonde data only consist of temperature, air pressure, and water vapor-related data up to 30 km, only the troposphere and lower stratosphere layers were updated. The AGT selected the best match from the six standard profiles for the upper atmosphere based on this lower atmosphere.

In Step 2, the total column concentration of CO_2_ was updated along with the scaling factors for N_2_O, CH_4,_ and four chlorofluorocarbons (CFCs). These updates adjusted those gases’ mixing ratios in the atmosphere. Additionally, the concentrations for N_2_O, CH_4_, O_3_, CO, NO_2_, SO_2,_ and the other uniformly mixed gases at all layers were updated based on the standard profile selected by the AGT. While the mixing ratios for these gases do not change with the profile selection, their concentrations vary based on each layer’s profile’s temperature and pressure.

In Step 3, the AQI data, which were considered localized, were used to modify the ground layer concentrations of O_3_, CO, NO_2_, and SO_2_. When these profile layers were modified, masks were used to delineate which layers were to be modified from those that should be determined by the standard profile selected by the AGT.

Lastly, in Step 4, the aerosols were adjusted if necessary. Unless cloud layers were being used, the troposphere’s and lower stratosphere’s aerosols were selected for spring/summer or fall/winter, and the upper stratosphere’s aerosols were modeled as meteoric dust. When an aerosol model was selected or the visibility range was adjusted, the concentration of small and large particles in the ground layers was adjusted. MODTRAN automatically scaled water-soluble substances and organic particle size depending on relative humidity when it computed atmospheric scattering. At this time, small (2.5 μm) and large (10 µm) particulate concentrations, commonly part of AQI data, were not correlated to a visibility range or aerosol model.

## 3. Longwave Infrared Results

### 3.1. Standard Profiles Comparison with Measured Data

**Figure 4:**
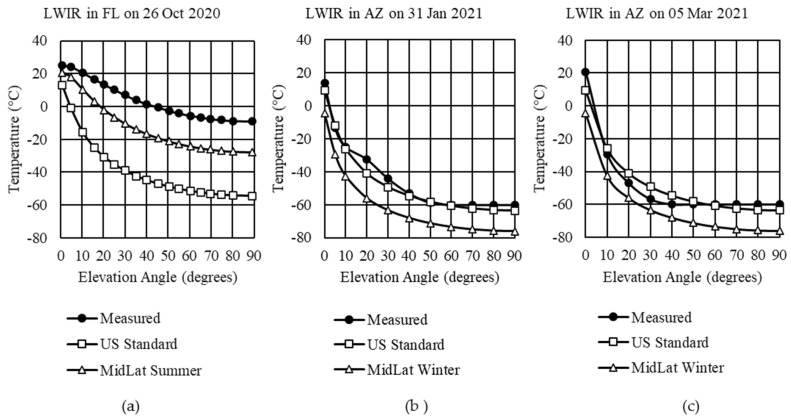
LWIR measured sky temperatures compared with MODTRAN’s standard profiles: (**a**) Example 1 in Cape Canaveral, Florida; (**b**) Example 2 in Tucson, Arizona; (**c**) Example 3 in Tucson, Arizona.

MODTRAN’s standard profiles provide results based on inputs developed from 1976. When using a standard or custom atmosphere profile, a date, time, and location affect the thermal emission that makes up the path radiance. For the first comparison, the only adjustment made was in Step 1, where a default atmosphere was selected. Gas concentrations were left at default values for Steps 2 and 3. Step 4 had its stratosphere aerosols adjusted by season and the default rural aerosol model for the ground layer. Figure 4 compares MODTRAN’s standard profiles for US Standard and MidLat summer or MidLat winter against the measured data for the three examples.

**Figure 5:**
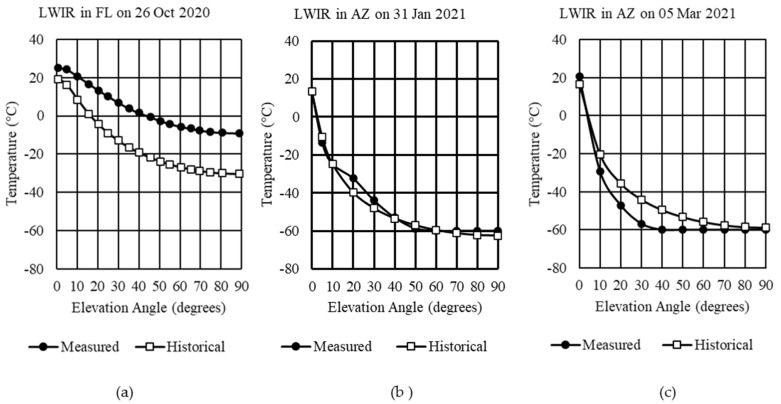
LWIR measured sky temperatures compared with historical averages of radiosonde data: (**a**) example 1 in Cape Canaveral, Florida; (**b**) example 2 in Tucson, Arizona; (**c)** example 3 in Tucson, Arizona.

In Figure 4a, there is a poor match of the measured sky temperatures in Florida with a predicted average temperature difference of 45.3 °C to the US Standard profile and 18.7 °C to the MidLat summer profile zenith. Arizona’s comparison on 31 January (Figure 4b) and 5 March 2021 (Figure 4c) show close agreement for the US Standard Profile but not the MidLat winter. It should be noted that the FLIR LWIR camera is limited to temperatures above −60 °C.

If, however, during Step 2, the total column concentrations were adjusted, minor changes to the results would be realized. We updated the volume mixing ratio for CO_2_, NO_2_, and scaling factors for uniformly mixed molecules with significant absorption in MWIR and LWIR with new global averages for 2020 and 2021. For example, the CO_2_ value increased from 399 ppmV in the default profile to 411.70 ppmV in October 2020, 414.90 ppmV in January 2021, and 416.32 ppmV in March 2021, according to Table 2. In the case of the LWIR in Florida, the average temperature at each elevation angle decreased by 0.30 °C for the US Standard profile and 0.14 °C for the MidLat summer profile, compared with those values in Figure 4. For the LWIR in Arizona on 31 January, a decrease of 0.20 °C and 0.25 °C for US Standard and MidLat winter, respectively, were obtained from MODTRAN. For 5 March, the decreases were 0.18 °C and 0.22 °C, respectively.

### 3.2. Historical Averages Comparison with Measured Data

In this comparison, historical averages of radiosonde data were used in Step 1, updated global gas concentrations in Step 2, AGT-selected gas concentrations for Step 3, and default aerosols for Step 4. During Step 1 of our method, historical averages of radiosonde data were used for the atmosphere profile from ground level up to the lower stratosphere (about 30 km). When the AGT was used to retrieve the historical averages, a better fit was obtained since now location, date, and time of day were being considered. Based on the latitudes of Florida and Arizona, each 2.5° × 2.5° grid would represent a land area of about 50,000 km^2^. The historical average was taken to the nearest quarter of the day from NOAA’s NCEP/NCAR Reanalysis database, as averaged from 1948 to present for each gridded area. The comparison between the measured sky temperatures and the historical averages is shown in Figure 5.

When atmosphere profiles depended on historical averages, results were close but did not correlate atmospheric effects sufficiently at all times. For example, as shown in Figure 5a, the Florida example still left a temperature difference of 5.74 °C at the horizon and 21.17 °C at zenith. In Arizona, for Examples 2 and 3, the January example (Figure 5b) provided better results than the March example (Figure 5c). Examples 2 and 3 still suffered from camera limitations that prevented measuring temperatures below −60 °C.

### 3.3. Daily Radiosonde Comparison with Measured Data

The same four steps were performed as in Section 3.2, except that daily radiosonde data were used instead of the historical averages in Step 1. Using the radiosonde data from the University of Wyoming’s weather database, even closer matches were obtained, as shown in Figure 5. For all examples, the radiosonde data for the 24:00 UTC launch were used, which correlated to different local times for each location. It should be noted that the radiosonde balloon’s ascent can take over two hours to reach an altitude of 35 km and have a lateral drift upwards of 300 km. Therefore, lower altitudes where measurements were taken closer to the launch time would provide more accurate adjustments to the atmosphere profile.

**Figure 6:**
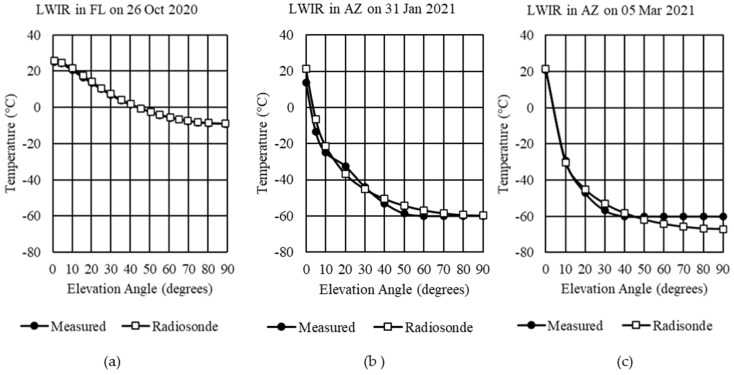
LWIR measured sky temperatures compared with daily radiosonde data: (**a**) Example 1 in Cape Canaveral, Florida; (**b**) Example 2 in Tucson, Arizona; (**c**) Example 3 in Tucson, Arizona.

As evident in Figure 6, radiosonde data significantly improved matching the measured equivalent blackbody sky temperatures to MODTRAN. For Example 1 (Figure 6a), the average difference was reduced to 0.38 °C from horizontal to zenith. Over the range of horizontal to 50° elevation angle, Example 2 (Figure 6b) had an average difference of 2.93 °C. Example 3 (Figure 6c) had an average difference of 1.48 °C over the range of horizontal to 40° elevation angle.

### 3.4. Daily Radiosonde with AQI Comparison with Measured Data

The last level of refinement was updating the ground-level atmosphere profile layers with AQI data provided by local weather stations for Step 3. These hourly and daily measurements would be only applicable to the lower portion of the troposphere up to about 3 km. By updating these layers, the sky path radiance was affected at all elevation angles.

The process of utilizing these AQI data required substituting concentrations of the corresponding gas molecules in the profiles. After the MODTRAN case’s atmosphere was adjusted with radiosonde data, the new values for O_3_, SO_2_, NO_2_, and CO concentrations up to 3 km replaced the corresponding values in the standard profile selected by the AGT. The comparison of average temperature differences for historical averages and radiosonde with AQI refinements to the MODTRAN case’s atmosphere is shown in Table 3.

## 4. Midwave Infrared Results

Compared with the LWIR FLIR T1020 camera, which has a spectral response from 7.5 µm to 14 µm, the MWIR TELOPS M1k has a spectral response from 2.2 µm to 5.5 µm.

The same steps shown in Figure 3 are applied to two sky measurements taken in Tucson, AZ, on 31 January 2021 at 19:00 local time and on 5 March 2021 at 18:30 local time.

### 4.1. Standard Profiles Comparison with Measured Data

**Figure 7:**
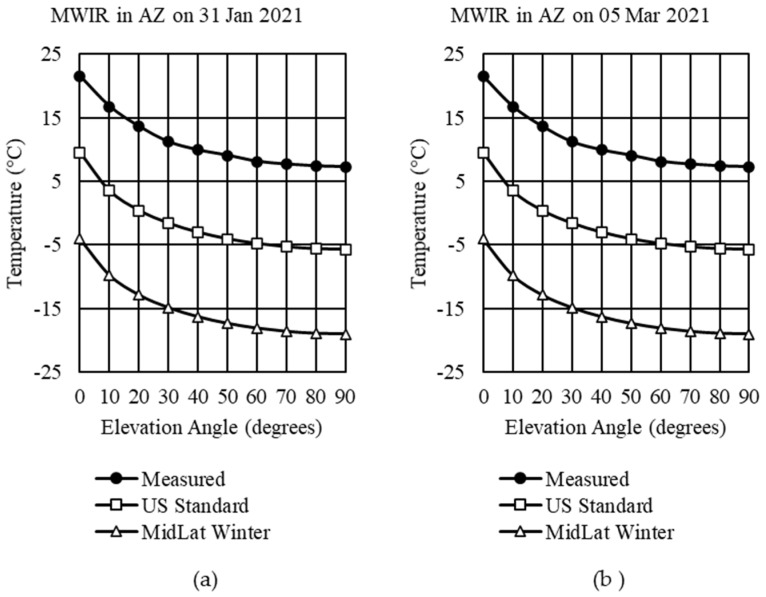
MWIR measured sky temperatures compared US Standard and MidLat winter: (**a**) Example 2 in Tucson, Arizona, on 31 January 2021; (**b**) Example 3 in Tucson, Arizona on 5 March 2021.

In Step 1, the default MODTRAN atmospheres, US Standard and MidLat winter, were selected while keeping all other parameters at their default values. The results showed significant differences between the measured sky data and the predictions, as shown in Figure 7.

When the total column concentration and scaling factors were updated in Step 2 according to Table 2, only minor corrections were seen. The temperatures for both US Standard and MidLat winter decreased by an average of 0.02 °C.

### 4.2. Historical Averages and Daily Radiosonde Comparison with Measured Data

**Figure 8:**
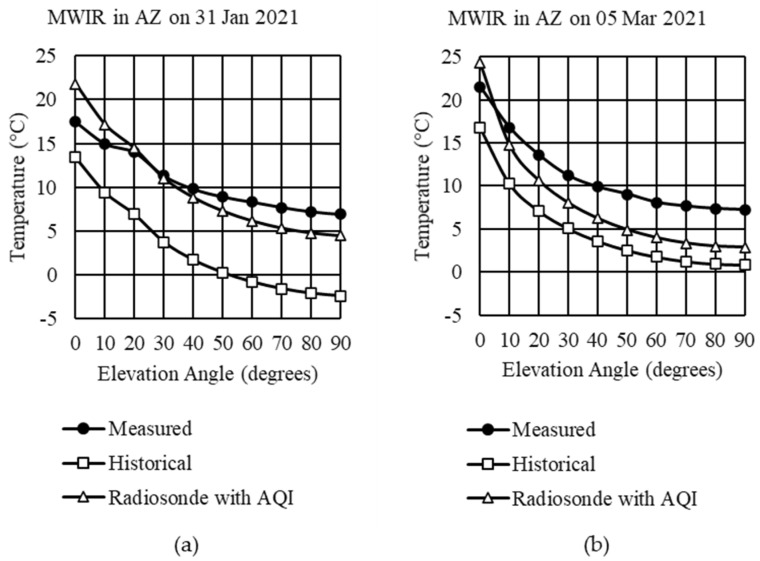
MWIR measured sky temperatures compared with historical averages daily radiosonde with AQI: (**a**) Example 2 in Tucson, Arizona on 31 January 2021; (**b**) Example 3 in Tucson, Arizona on 5 March 2021.

The following comparison used the historical averages and daily radiosonde in Step 1. For both examples shown in Figure 8, the total column concentrations were updated in Step 2. In Step 3, the AGT selected the profiles for the gas concentrations, and AQI data were included for the ground layer. When using historical 4X daily averages, there was a considerable underestimation of the path radiance, most likely due to the averaging over six hours. While not as good as a match as achieved in the LWIR, the 3–5 °C obtained using daily radiosonde and AQI information was significantly better than the discrepancies seen using one of the default profiles.

There are several possible causes for why MWIR results did not match as well as LWIR. First, there was more scattering in MWIR than LWIR. When using MODTRAN, we only considered single scattering predictions compared with multiple scattering. Second, the ground surface was modeled with zero reflectance. This was a more accurate assumption for LWIR, as dirt, sand, and vegetation have emissivities over 0.9. These same materials would have lower emissivities in MWIR, adding solar reflections from the ground into the radiation transfer model. Third, radiosonde data collection occurred over two hours as the balloon ascended. The measured data were taken over less than 5 min, meaning that the higher altitude radiosonde data did not represent current conditions as lower values. Since path radiance was greater in MWIR than LWIR, measurements in MWIR may be more sensitive to changes in temperature, pressure, and water vapor concentration over short periods of time. Finally, the ability to estimate temperature depends upon the sensor spectral response. The spectral response provided by the manufacturer is always an approximation. We believe that the provided MWIR sensor spectral response may not match our camera’s response, leading to the temperature discrepancy.

## 5. Discussion

Depending on the application, different amounts of custom inputs may be needed for modeling the atmosphere. As a coarse method, selecting one of the six standard profiles is only be applicable when the atmosphere and weather conditions are close to the annual averages. The extreme difference between the standard atmosphere and measured data was especially evident in Example 1 for Florida. There was almost a 45 °C difference in the LWIR between measured data and the US Standard atmosphere profile when viewing the sky at the zenith. This difference was not as dramatic in the MWIR but significant enough that it should not be used to model a drone’s detectability in a specific geographic location or time of the year.

Historical averages provide a suitable atmosphere profile for predicting performance in a specific geographical location. With radiosonde data available every six hours, seasonal or daytime/nighttime comparisons can be performed. In Table 3, the results showed an average difference between 2.44 °C to 18.29 °C, which may be sufficient for modeling intraday extremes. However, since the averages from NOAA’s NCEP/NCAR Reanalysis database were from 1948 to the present, the values may not represent global trends to atmospheric composition in recent years. Using historical averages may be more appropriate when there is no radiosonde collection station nearby.

While historical averages are adequate for predicting a specific camera’s performance for detecting small aerial targets, daily radiosonde is most appropriate for analyzing data collection. As shown in the three examples in Figure 6, when the radiosonde data were from the exact geographic location and time that measurement data were taken, an extremely close match could be obtained. In the Florida example, the measured data and the MODTRAN prediction had a 0.38 °C average match from horizon to zenith. If the background path radiance matched the prediction, the atmosphere profile would likely be correct for obtaining atmospheric transmission values using MODTRAN or similar modeling tools.

The MWIR examples did not provide as close a match with daily radiosonde but were still better than using historical averages. There are several likely causes for this. First, the higher equivalent blackbody temperatures over the entire elevation range show that there was more path radiance being generated. This was further exacerbated because the TELOPS MWIR camera was not fitted with a CO_2_ notch filter; CO_2_ is a significant source of path radiance in MWIR. Therefore, when global averages were used to adjust the mixing ratio, those quantities may not have accurately represented local conditions where data were collected. There could also have been horizontal variations that could not be captured in a MODTRAN atmosphere profile.

Updating total column concentrations and scale factors for uniformly mixed gases did not have a significant impact. As reported in Section 3.1, updating present-day values resulted in an average change of around 0.2 °C. The same conclusion could be drawn from incorporating AQI data for the ground level (0–3 km) atmosphere profile layers. Including local AQI data for O_3_, SO_2_, NO_2_, and CO concentrations improved the match for Florida but worsened it for Arizona. The most likely reason for this is that the AQI values were low in rural areas; this would not be the case in urban or industrial areas.

While MODTRAN adjusts the concentration and size of the aerosols based on air pressure and humidity, local weather data for aerosols are vague and do not readily translate to MODTRAN inputs. In this study, the concentration of aerosols at the ground level for all three examples relied on the rural profile, as the measured data were taken outside urban areas. In all cases, we found that the maximum visibility was reported as “greater than 10 miles (16 km)”, which prevented using a longer range with any confidence in Step 4 of our process.

## 6. Conclusions

Refining one of the six standard atmosphere profiles is a necessary step when evaluating the performance of any long-range infrared imaging system. In general, when determining the operational limitations of a system or performing predictive modeling of a small aerial target against a sky background, at the minimum, historical averages should be used to generate a custom atmosphere profile. However, a more customized atmosphere profile should be used when specific imagery is being analyzed or tools such as adaptive contrast enhancement. Daily radiosonde data should be used in these cases, and efforts should be made to interpolate the radiosonde data for the time of day and location. Even when a non-radiometric calibrated system is used, when radiosonde data are used for creating a custom atmosphere in MODTRAN, you can infer the sky background characteristics with a high level of confidence. While we showed that updating atmospheric concentrations affects sky path radiance results, new molecular concentrations have only a minor impact on broadband infrared imaging in a clear sky environment. AQI data are a convenient source for updating ground-level atmosphere composition. However, it would be limited to specific applications such as highly polluted urban areas or narrowband infrared camera systems.

## Figures and Tables

**Table 1:** Major absorption peaks of gases tracked by NOAA Global Monitoring Laboratory (GML) and the US Environmental Protection Agency’s (EPA) Air Quality Monitoring Stations [23].

Component	Absorption Peaks [µm]	Component	Absorption Peaks [µm]
Carbon Dioxide	4.3, 13.8	Ozone	4.2, 9.6
Nitrous Dioxide	4.5, 7.7	Carbon Monoxide	4.6
Methane	3.4, 7.6	Sulfur Dioxide	7.5
Water Vapor	6.3, greater than 10	Nitrogen Dioxide	7.7

**Table 2:** Updated atmosphere values for MODTRAN: GML—NOAA Global Monitoring Laboratory; AGT—Atmosphere Generator Toolkit; MODTRANv6—MODTRAN Version 6 User Manual; US EPA—US Environmental Protection Agency Air Quality.

Component	Source	Example 1 Florida 26 October 2020	Example 2 Arizona 31 January 2021	Example 3 Arizona 5 March 2021
*Total Column*				
CO_2_ [ppmV]	GML	411.72	414.90	416.34
Water vapor [ppmV]	AGT	Radiosonde	Radiosonde	Radiosonde
*Scale Factors*				
N_2_O	GML	1.104	1.104	1.104
CH_4_	GML	1.111	1.111	1.111
CFC-11	MODTRANv6	1.674	1.674	1.674
CFC-12	MODTRANv6	2.170	2.170	2.170
CFC-113	MODTRANv6	3.758	3.758	3.758
CCl_4_	MODTRANv6	0.629	0.629	0.629
*Ground Level*				
O_3_ [ppbV]	US EPA	26.0 ^1^	36.3	39.0
CO [ppbV]	US EPA	300 ^1^	132	200
NO_2_ [ppbV]	US EPA	4.30 ^1^	4.01	10.2
SO_2_ [ppbV]	US EPA	3.40 ^1^	0.14	0.10

^1^ Nearest station value is from Orange County, Florida.

**Table 3:** The average temperature difference between measured data and atmosphere profiles over elevation ranges for LWIR.

Location (Elevation Range)	Historical Averages	Radiosonde	Radiosonde with AQI
Example 1 Florida (0°–90°)	18.29 °C	0.38 °C	0.34 °C
Example 2 Arizona (0°–50°)	2.44 °C	4.38 °C	4.41 °C
Example 3 Arizona (0°–40°)	8.01 °C	1.48 °C	1.69 °C

## Data Availability

The measured sky data (Examples 1, 2, and 3) presented in this article are available in the Mathworks Central File Exchange, https://www.mathworks.com/matlabcentral/fileexchange/94680-sky-temp-wrapper-for-modtran6 (accessed on 15 June 2021). Copies of daily radiosonde corresponding to each example are publicly available at the Department of Atmospheric Science, University of Wyoming, http://weather.uwyo.edu/upperair/sounding.html (accessed on 15 June 2021). Global averages for updated atmospheric gas concentrations as provided in Table 2 are publicly available at https://gml.noaa.gov/ccgg/trends/ (accessed on 15 June 2021).

## Supplementary Materials

The following are available online at https://www.mathworks.com/matlabcentral/fileexchange/94680-sky-temp-wrapper-for-modtran6 (accessed on 15 June 2021): TemplateMaker.mlx for creating MODTRAN custom atmosphere profiles; Modtran_for_Matlab.mlx and supporting functions for computing equivalent blackbody sky temperatures with custom atmospheres; measured sky data for Examples 1, 2, and 3.
